# Uniportal thoracoscopic plication of diaphragmatic eventration: loop needle technique for better visualization

**DOI:** 10.1093/icvts/ivae164

**Published:** 2024-09-30

**Authors:** Ryosuke Kumagai, Shinsaku Kabemura, Fumitsugu Kojima, Toru Bando

**Affiliations:** Department of Thoracic Surgery, St Luke’s International Hospital, Tokyo, Japan; Department of Thoracic Surgery, St Luke’s International Hospital, Tokyo, Japan; Department of Thoracic Surgery, St Luke’s International Hospital, Tokyo, Japan; Department of Thoracic Surgery, St Luke’s International Hospital, Tokyo, Japan

**Keywords:** Diaphragmatic eventration, Knifeless endostapler, Uniportal thoracoscopic surgery, Diaphragm plication, Dynamic magnetic resonance imaging, Paradoxical movement

## Abstract

Symptomatic unilateral diaphragmatic eventration require surgical intervention. A 56-year-old woman complained of dyspnoea on exertion and was noted to have left diaphragm elevation on chest radiographs. Dynamic magnetic resonance imaging showed paradoxical movement of the left diaphragm. We performed diaphragmatic plication by uniportal thoracoscopy with knifeless endostaplers and a loop needle device. Her symptoms significantly improved immediately after the operation, and this condition had been maintained for 6 months. We thus suggest this minimally invasive technique as an easy and safe method for diaphragmatic plication.

## INTRODUCTION

In adult patients with unilateral diaphragm diaphragmatic eventration (UDE), plication of the diaphragm was reported to provide long-lasting improvements in symptoms [1]. We described a case in which we devised a minimally invasive surgical technique, uniportal thoracoscopic plication, using a knifeless endoscopic linear stapler and a loop needle device.

## ETHICAL STATEMENT

For this case report, approval from the Institutional Review Board was waived by our hospital. Written informed consent was obtained from the patient.

## CASE PRESENTATION

**Figure 1:**
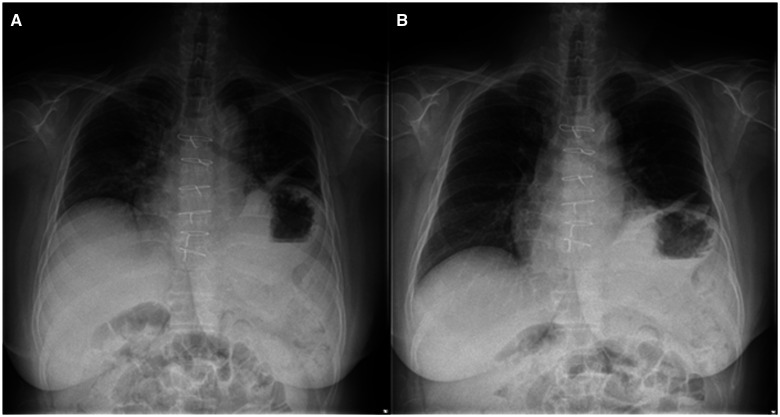
Preoperative chest radiograph. The left hemidiaphragm is markedly elevated and does not move with (**A**) exhalation and (**B**) inhalation, which shows downward movement of the right diaphragm.

A 56-year-old woman presented with severe dyspnoea on exertion, which started as gradual worsening after coronary artery bypass graft 2 years ago. Chest radiograph showed a markedly elevated and paralyzed left hemidiaphragm (Fig. 1). Dynamic magnetic resonance imaging displayed poor and paradoxical movement of the left diaphragm, compared with that of the right diaphragm (Video 1).

**Figure 2:**
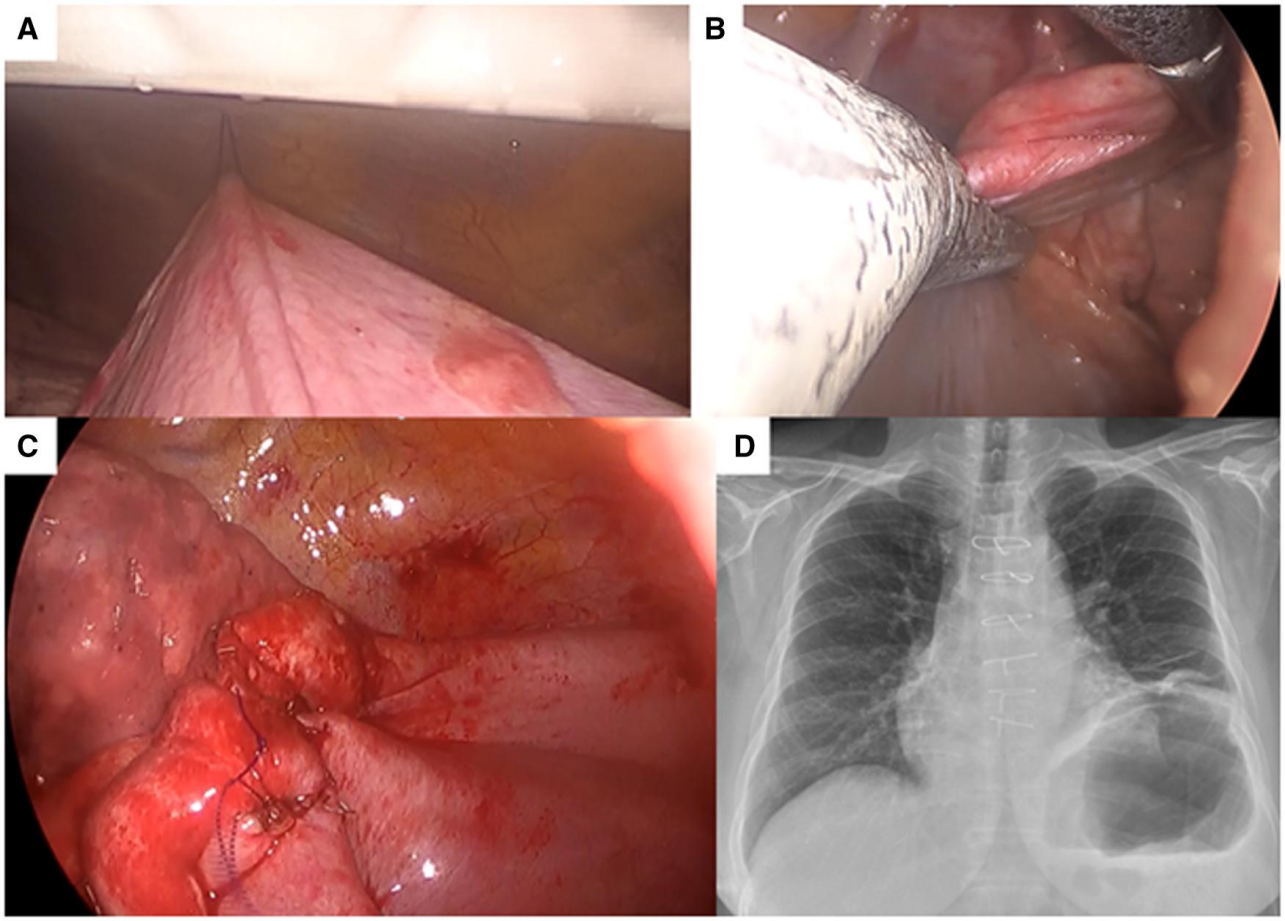
Surgical images. (**A**) A traction thread held by the loop needle device can be used to fix or change the diaphragm position through a temporal puncture access to achieve adequate visualization and working space without the need for additional incisions. (**B**) The diaphragm is plicated with a knifeless endoscopic linear stapler and (**C**) sutured to create the desired tension and increase the plication strength. (**D**) Six months postoperative chest radiograph.

Video-assisted thoracoscopic surgery (VATS) was performed with the patient placed in the lateral decubitus position (Video 2). A single 3-cm lateral incision was made in the 7th intercostal space, lateral to the middle axillary line. First, we made a small incision on the diaphragm to inspect and ensure that there were no other adhesions between the diaphragm and intraabdominal organs. Thereafter, a portion of the redundant diaphragm was pulled and lifted with an endograsper while being plicated with a knifeless endoscopic linear stapler (ENDOPATH ENDOCUTTER ETS45, J&J Ethicon, Tokyo, Japan). A loop needle device (LAPA-HER-CLOSURE; Hakko, Co., Ltd., Tokyo, Japan) was used to appropriately fix and change the diaphragm position through punctures on the 7th intercostal space lateral to the posterior axillary line, 9th intercostal space lateral to the posterior axillary line and 4th intercostal space lateral to the anterior axillary line. After repeating the abovementioned procedure 3 times, we sutured the diaphragm by 3–0 PDS to create the desired tension and increase the strength of the plications (Fig. 2). A nonwoven polyglycolic acid felt was used to cover and reinforce the plicated diaphragm. The operative time was 124 min. The patient was discharged on postoperative day 4. For 6 months after the procedure, she had been tolerating exercise well, with improvements in the following: (i) Modified Medical Research Council Dyspnoea Scale from grade 3 to 1; (ii) pulmonary function parameters, such as forced vital capacity (from 1.36 to 1.54 l) and forced expiratory volume in 1 s (from 1.13 to 1.26 l/s); (iii) absence of paradoxical movement of the left diaphragm on dynamic magnetic resonance imaging (Video 3); and (iv) 500 m without desaturation on 6-min walk test (there was no preoperative value because the patient was dyspnoeic and refused).

## DISCUSSION

Patients with chronic dyspnoea caused by UDE were reported to significantly benefit from diaphragm plication [2]. Although not yet established, phrenic nerve reconstruction may be the ideal treatment for UDE.

Richard claimed that the principle of management is to keep the diaphragm nearly flat and taut [2]. In the present case, however, keeping the diaphragm taut improved her symptoms, although the diaphragm position was not flat. The most important factor when treating UDE is to fixate the diaphragm and prevent paradoxical movement; in this case, uniportal VATS was sufficient to accomplish the purpose. Compared with multiport VATS, uniportal VATS was reported to have the same surgical outcome [3], a significantly lower incidence of post-thoracotomy pain syndrome, and result in faster recovery and better cosmesis [4].

Previously, we used to suture the diaphragm with a thread during multiport VATS. To facilitate easier uniportal thoracoscopic plication, we utilize a combination of 2 useful devices. First is the use of knifeless endostaplers, which was initially reported by Moon *et al*. [1] in a case that underwent multiport VATS with multiple plications to create a tense diaphragm. Indeed, this technique is useful for reinforcing the diaphragm and enables easy suturing. Moreover, additional suturing can be easily performed if diaphragm tension is needed. A recent review mentioned studies on uniportal VATS but none on a knifeless endostapling device [5].

Another useful instrument is a loop needle device, which has a wire loop that can hold and release a thread, allowing easy fixation of the diaphragm by an assistant and repeatable positional changes to maintain proper visualization without the need for additional incisions. There had been no reports on the use of a loop needle device to address the issue on visibility, which is crucial in uniportal VATS.

## CONCLUSIONS

This new uniportal thoracoscopic plication, which combines 2 useful devices, was a safe and easy method for plication of UDE.

## Data Availability

The data underlying this article will be shared on reasonable request to the corresponding author.
